# Benefits of Whole-Body Vibration, as a Component of the Pulmonary Rehabilitation, in Patients with Chronic Obstructive Pulmonary Disease: A Narrative Review with a Suitable Approach

**DOI:** 10.1155/2016/2560710

**Published:** 2016-04-14

**Authors:** Danubia Sá-Caputo, Cintia Renata Gonçalves, Danielle Soares Morel, Eloá Moreira Marconi, Patrícia Fróes, Rogério Rufino, Cláudia Henrique Costa, Agnaldo José Lopes, Adriano Arnóbio, Nasser Ribeiro Asad, Pedro Jesus Marin, Trentham Furness, Mario Bernardo-Filho

**Affiliations:** ^1^Programa de Pós-Graduação em Fisiopatologia Clínica e Experimental, Universidade do Estado do Rio de Janeiro, Rio de Janeiro, RJ, Brazil; ^2^Programa de Pós-Graduação em Ciências Médicas, Universidade do Estado do Rio de Janeiro, Rio de Janeiro, RJ, Brazil; ^3^Universidade Potiguar, Natal, RN, Brazil; ^4^Departamento de Especialidades Médicas (Pneumologia e Tisiologia), Faculdade de Ciências Medicas, Universidade do Estado do Rio de Janeiro, Rio de Janeiro, RJ, Brazil; ^5^Departamento de Biofísica e Biometria, Instituto de Biologia Roberto Alcantara Gomes, Universidade do Estado do Rio de Janeiro, Rio de Janeiro, RJ, Brazil; ^6^Laboratory of Physiology, European University Miguel de Cervantes, Valladolid, Spain; ^7^CYMO Research Institute, Valladolid, Spain; ^8^Departmento de Clínica Médica, Universidade do Estado do Rio de Janeiro, Rio de Janeiro, RJ, Brazil; ^9^School of Nursing, Midwifery and Paramedicine, Australian Catholic University, Fitzroy, VIC, Australia

## Abstract

*Background*. Appropriate management, including pulmonary rehabilitation, associated with correct diagnosis of chronic obstructive pulmonary disease (COPD) in patients can contribute to improving clinical conditions of these patients. Physical activity is recommended for COPD patients. Whole-body vibration (WBV) is a modality of physical activity. Putting together the biological effects and safe use of WBV, it may be a potentially feasible intervention to add to pulmonary rehabilitation. The purpose of this investigation was to systematically review studies regarding the effects of WBV, as a component of the pulmonary rehabilitation, in patients with COPD.* Results*. A total of six publications met inclusion for review. There was evidence to support the beneficial use of WBV to improve functional performance of the lower limbs and quality of life. However, the appropriateness of and descriptors of WBV methods were poorly described.* Conclusions*. The results of this review support the use of WBV as a component of pulmonary rehabilitation to assist management of patients with COPD. However, future research should examine the dose-response curve and optimal dosing regimen of WBV according to standard reporting recommendations for people with COPD. Such an approach will allow comparison among studies and the potential of meta-analysis of randomized controlled trials.

## 1. Background

A disease of the lungs, chronic obstructive pulmonary disease (COPD), is a preventable and/or treatable respiratory disease. It can be characterized by progressive airflow limitation [1] and chronic inflammatory response to noxious particles or gases [2]. However, the progressive and incurable nature of COPD remains a major public health problem across the developing and developed world [2]. The disease is a significant contributor to global morbidity and mortality, accounting for more than 3 million deaths in 2012 [3]. In the United States of America alone, COPD was the third-ranked cause of mortality, responsible for more than 120,000 annual deaths [4]. Furthermore, COPD is associated with comorbid conditions such as skeletal muscle dysfunction and cardiovascular diseases [5]. In addition, the severity of the disease and associated exacerbations may require repeated clinical evaluations, treatments, and inpatient admission [1].

Pulmonary rehabilitation can be a nonpharmacological intervention that is part of clinical management of patients with chronic respiratory disease who remain symptomatic or continue to have decreased function despite standard medical therapy [2, 6–8]. Among people with COPD, pulmonary rehabilitation can reduce dyspnoea and fatigue [6] and improve peripheral skeletal muscle function [2, 7] and quality of life [9, 10] and may prolong survival [11]. As a component of pulmonary rehabilitation, physical activity is recommended to all patients with a diagnosis of COPD and may improve exercise tolerance and performance of activities of daily living [2]. However, given that aerobic conditioning [12] and resistance training [13] are associated with high levels of perceived dyspnoea, fear of breathlessness can lead to reduced participation in physical activity [14, 15] and the so called dyspnoea spiral [16]. As such, the use of peripheral muscle training in the management of patients with COPD involves careful consideration given the potential for exacerbations, the risk of acute dyspnoea or hypoxemia during resistance training [13, 17], and/or aerobic conditioning [18–20]. Due to the necessity of physical activity to the COPD patient and the potential limitations of both aerobic conditioning and resistance training, other modalities of exercise should be examined.

As a component of pulmonary rehabilitation, whole-body vibration (WBV) is emerging as a potentially beneficial modality of physical activity for people with COPD [21–27]. Whole-body vibration has also emerged among other populations with suboptimal health such as fibromyalgia [28], cystic fibrosis [29–31], and multiple sclerosis [32, 33]. As a mode of physical activity, WBV may be performed when a person either (a) stands stationary on a base of the platform or (b) performs movements while standing, sitting, or lying on an oscillating/vibratory platform such as flexion and extension of the lower limbs [34–37]. It is speculated that the effect of WBV on the musculoskeletal system is to produce changes in the length-tension relationship moderated within the muscle spindle and may elicit a tonic vibration reflex [36, 38, 39] and subsequently improve the performance of skeletal muscles of the lower limbs [21].

A recent systematic review of COPD and WBV aimed to report on outcome measures of functional performance: the 6-minute walk test, the sit-to-stand test, peak knee extension force, and quality of life [40]. Despite a small number of randomized controlled trials, preliminary evidence emerged to support the use of WBV to improve the aforementioned measures of functional performance of people with COPD [40]. However, evidences about the specific frequency, intensity, type, duration, and gravitational properties of the vibration platform and WBV are yet to be described that may enhance peripheral muscle training for people with a diagnosis of COPD. Some recommendations by the International Society of Musculoskeletal and Neuronal Interactions (ISMNI) about WBV interventions include reporting [41] (1) the vibration device and brand, (2) direction, frequency, peak-to-peak displacement, gravitational forces, and accuracy, and (3) evaluation of skidding and foot position.

As evidence is supporting the beneficial use of WBV for people with COPD [40], clinicians should be able to prescribe WBV as a mode of peripheral muscle training based on best available evidence. However, there is a total absence of how WBV should be prescribed to improve peripheral muscles of the lower limbs of people with COPD. Therefore, the aim of this narrative review was to advance the seminal work of Gloeckl et al. (2015) [40] by describing the methods of WBV exercise for people with COPD using ISMNI recommendations and describe evidence based on National Health and Medical Research Council hierarchy of evidence (NHMRC, 2003–2007) [42].

## 2. Methods

### 2.1. Search Strategy

Three reviewers independently accessed bibliographical databases through the* Universidade do Estado do Rio de Janeiro*. Searches were performed in the PubMed (MEDLINE), Scopus, Science Direct, and PEDro databases on February 9, 2015, each with the keywords “Chronic obstructive pulmonary disease” OR “COPD” AND “whole-body vibration” OR “WBV”. The review was performed with PRISMA guidelines [43].

### 2.2. Inclusion and Exclusion Criteria

To be included for review, all studies investigating effects of WBV in persons with COPD needed to be conducted as a randomized controlled trial or single group experimental studies with crossover designs. Only studies published in English were considered for inclusion. Studies were reviewed if they included participants with COPD and who performed static or dynamic exercises on a WBV platform and the method of WBV was clearly described. Inclusion for review was based on consensus among three reviewers. Data were independently abstracted by the same three reviewers and disagreements were resolved by majority consensus. Studies/papers were excluded if they were review articles, replies, editorials, trial protocols, books, or chapters.

### 2.3. Level of Evidence of the Selected Papers

The included studies were classified according to the National Health and Medical Research Council hierarchy of evidence (NHMRC, 2003–2007) [42] (see Table 1). Each article was assigned to one reviewer and cross-checked by a second reviewer and where there was disagreement a third party was consulted and the issue discussed until consensus was reached.

## 3. Results

**Figure 1:**
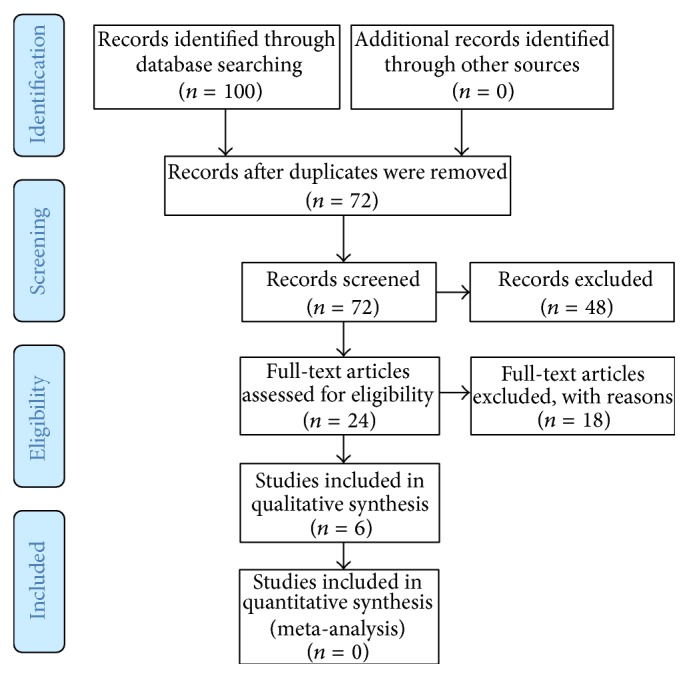
PRISMA flowchart.

Of thousands of papers identified (see Table 2), a total of 100 were screened for review. A total of six papers met the inclusion criteria. A meta-analysis was inappropriate due to a vast difference of intervention group and control group interventions among the level of evidence II studies (see Figure 1). The descriptions and level of evidences of six reviewed studies are shown in Table 3. The level of evidence was Level II [23, 26, 27] and Level III-1 [22, 24, 25]. The degree to which the six reviewed papers met ISMNI recommendations is shown in Table 4.

### 3.1. Level of Evidence II and ISMNI Recommendations for WBV Interventions

At this level of evidence, varying WBV interventions improved QoL and functional performance of the lower limbs of people with COPD [23, 26, 27]. However, the interventions varied relative to the direction of the vibration platform, the vibration frequency, peak-to-peak displacement, and gravitational forces (see Table 4). The validity of each vibration platform was not established (i.e., frequency and displacement) nor was skidding or the position of the feet upon the vibration platform.

### 3.2. Level of Evidence III-1 and ISMNI Recommendations for WBV Interventions

At this level of evidence, WBV improved functional performance of the lower limbs [22, 24, 25] and QoL [22] of people with COPD (see Table 4). Two studies met the ISMNI recommendations [24, 25]. One study did not report vibration direction or test for accuracy and skidding.

## 4. Discussion

The major finding of this study was the benefit of WBV, as a component of pulmonary rehabilitation, for people with COPD. Specifically, evidence was found to support the use of WBV to improve functional performance of the lower limbs and QoL (see Table 5). The results of the current study support the seminal work of Gloeckl et al. (2015) [40]. However, despite evidence to support the use of WBV for people with COPD, the results of the current review revealed that reporting WBV methods are poorly disclosed.

By accepting manufacture claims of vibration frequency and peak-to-peak displacement rather than ascertaining vibration parameters by using accelerometers, gravitational forces cannot be confidently described [41]. Furthermore, if skidding is not assessed, the parameters of the vibration study that the participant is subjected to can no longer be defined [Rauch]. Skidding occurs when the feet lose contact with the vibration platform with increasing gravitational forces [34]. Only two studies of lower evidence [24, 25] could confirm the vibration parameters for the participants with COPD. As such, despite levels of evidence II and III-1 supporting the use of WBV for people with COPD, clinicians remain without a clear method of how WBV should be prescribed.

Whole-body vibration has been used without exacerbating people with COPD. The lowest frequency of the vibrations generated in the platforms was about 20 Hz [23]. Although all the authors of the investigations selected in this narrative revision [22–27] have suggested that the conditions of the protocols are without problems to the COPD patient, it would be interesting to use protocols with frequencies lower than 20 Hz. Lower vibration frequency has been beneficially used among patients with Parkinson's disease with 3 Hz, 6 Hz, and 9 Hz [44] and with fibromyalgia (12.5 Hz) [28]. The benefit of lower vibration frequency for people with COPD is that the risk of damaging fragile bone of older adults may be mitigated [45]. Subsequently, the appropriateness of prescribed doses of WBV should be at the forefront of clinicians as people with COPD are known to have lower bone mineral density compared with healthy matched controls [46, 47].

In general, the studies included in the current review reported benefits without important clinical complication to the patient with COPD. These findings are highly desirable and support the use of WBV as a component of the pulmonary rehabilitation. Whole-body vibration was reported as effective and feasible interventions [27] which does not exacerbate perceived dyspnoea [24, 25] and may safely improve clinical parameters of the patient with COPD [22].

## 5. Conclusions

The results of this review support the use of WBV as a potentially important component of pulmonary rehabilitation to assist management of patients with a diagnosis of COPD. Furthermore, WBV has been feasible and safely completed by patients with COPD. However, future research should examine the dose-response curve and optimal dosing regimen of WBV according to ISMNI recommendations for people with COPD. Such an approach will allow comparison among studies and the potential of meta-analysis of randomized controlled trials.

## Figures and Tables

**Table 1:** NHMRC levels of evidence relevant to the review inclusion criteria.

Level	Intervention	Diagnostic accuracy
I	A systematic review/meta-analysis of Level II studies	A systematic review of Level II studies

II	A randomized controlled trial	A study of test accuracy with an independent, blinded comparison with a valid reference standard, among consecutive persons with a defined clinical presentation

III-1	A pseudorandomized controlled trial	A study of test accuracy with an independent, blinded comparison with a valid reference standard, among nonconsecutive persons with a defined clinical presentation

**Table 2:** Number of publications identified.

Search	Keywords	PubMed	Scopus	PEDro	Science Direct
1	“Chronic obstructive pulmonary disease” OR “COPD”	97,248	70,969	1559	108,463

2	“Whole body vibration” OR “WBV”	1,257	2,626	167	2,738

Combined 1 and 2		16	25	5	54

**Table 3:** Level of evidence and outcomes of reviewed papers.

Level of evidence	Authors	Sample	Protocols and outcome measures	WBV findings
II	Greulich et al., 2014 [23]	*N* = 40 (26 males and 14 females) IG *n* = 20 IG age = 66 ± 10 years CG *n* = 20 CG age = 70 ± 10 years	IG: standard physiotherapy with WBV CG: standard physiotherapy Duration: during inpatient admission Outcomes: CRT, 6MWT, QoL, and serum markers	Improved CRT, 6MWT, and QoL Increased expression of the TFPP receptor gamma coactivator-1-*α* and levels of irisin Decreased serum interleukin-8

II	Pleguezuelos et al., 2013 [26]	*N* = 51 males IG *n* = 26 IG age = 73 ± 14 years CG *n* = 25 CG age = 74 ± 10 years	IG: WBV CG: lifestyle education Duration: 6 weeks Outcomes: IKFET lower limb performance, 6MWT, and pulmonary muscular assessment with MIP and MEP	No differences for IKFET Improved 6MWT, MIP, and MEP

II	Gloeckl et al., 2012 [27]	*N* = 72 (37 males and 35 females) IG *n* = 36 IG age = 64 ± 11 years CG *n* = 36 CG age = 65 ± 7 years	IG: WBV with dynamic squats CG: dynamic squats Duration: 3 weeks Outcomes: 6MWT, sit-to-stand test, and QoL	Improvement in 6MWT, sit-to-stand test No difference between groups for QoL

III-1	Braz Júnior et al., 2015 [22]	*N* = 11 (8 males and 3 females) Age: 63 ± 9 years Design: crossover with washout	IG: WBV CG: No intervention Duration: 12 weeks Outcomes: 6MWT, IPE, and QoL	Improved 6MWT and QoL No difference among groups for IPE

III-1	Furness et al., 2014 [24]	*N* = 16 (12 males and 4 females) Age: 72 ± 9 years Design: crossover with washout	IG: WBV CG: SWBV Duration: 6 weeks Outcomes: Borg CR-10, heart rate, saturation of oxygen, TUG test, 5-chair stand test, and gait velocity	No exacerbations were reported during the WBV or SWBV interventions. After improved TUG test, 5-chair stand test and gait velocity No meaningful difference among groups for Borg CR-10, heart rate, and saturation of oxygen

III-1	Furness et al., 2013 [25]	*N* = 17 Age: 69 ± 8 years Design: crossover with washout	IG: WBV CG: SWBV Duration: 1 session Outcomes: Borg CR-10, heart rate, and saturation of oxygen	No meaningful differences among groups

IG: intervention group; CG: control group; CRT: chair rising test; 6MWT: 6-minute walk test; QoL: quality of life; IKFET: isokinetic knee flexor/extensor testing; MIP: maximum inspiratory pressure; MEP: maximum expiratory pressure; IPE: index of perceived exertion; SWBV: sham whole-body vibration.

**Table 4:** Descriptors of WBV based on ISMNI recommendations.

Authors	Vibration device	Vibration direction	Vibration frequency	Peak-to-peak displacement	Gravitational force	Accuracy	Skidding	Foot position
Braz Júnior et al., 2015 [22]	Power Plate	Not stated	35 Hz 35 Hz	1 mm 2 mm	2.46 g 4.92 g	Not assessed	Not assessed	200 mm apart
Greulich et al., 2014 [23]	Galileo	Side alternating	12 Hz 26 Hz 26 Hz	3 mm 4 mm 6 mm	0.86 g 5.43 g 8.15 g	Not assessed	Not assessed	Not stated
Furness et al., 2014 [24]	Amazing Super Health	Side alternating	25 Hz	2 mm	2.52 g	Assessed	Assessed	200 mm from axis of rotation
Furness et al., 2013 [25]	Amazing Super Health	Side alternating	25 Hz	2 mm	2.52 g	Assessed	Assessed	200 mm from axis of rotation
Pleguezuelos et al., 2013 [26]	Fitybe excel pro	Vertical	35 Hz	4 mm	9.85 g	Not assessed	Not assessed	Not stated
Gloeckl et al., 2012 [27]	Galileo	Side alternating	24 Hz 25 Hz 26 Hz	6 mm^*∗*^	Cannot be calculated	Not assessed	Not assessed	Not stated

^*∗*^Peak-to-peak amplitude reported.

**Table 5:** Statement of evidence for WBV interventions among people with COPD.

Statement	Level of evidence
WBV improves performance of field tests that simulate activities of daily living (e.g., 6MWT and sit-to-stand test)	II and III-1
WBV improves serum markers associated with COPD	II
WBV may improve quality of life	II and III-1
WBV does not add clinically meaningful stress on the cardiorespiratory system	III-1

## Abbreviations

CG: Control group
COPD: Chronic obstructive pulmonary disease
GOLD: Global Initiative for Chronic Obstructive Lung Disease
IG: Intervention group
IKFET: Isokinetic knee flexor/extensor testing
IPE: Index of perceived exertion
ISMNI: International Society of Musculoskeletal and Neuronal Interactions
MEP: Maximum expiratory pressure
MIP: Maximum inspiratory pressure
6MWT: Six-minute walk test
NHMRC: National Health and Medical Research Council
PEDro: Physiotherapy Evidence Database
QoL: Quality of life
SWBV: Sham whole-body vibration
TFPP: Transcription factor peroxisome proliferator
TUG: Timed up and go test
WBV: Whole-body vibration.
